# Four Distinctions for the Auditory “Wastebasket” of Timbre^1^

**DOI:** 10.3389/fpsyg.2017.01747

**Published:** 2017-10-04

**Authors:** Kai Siedenburg, Stephen McAdams

**Affiliations:** ^1^Centre for Interdisciplinary Research in Music Media and Technology, Schulich School of Music, McGill University, Montreal, QC, Canada; ^2^Department of Medical Physics and Acoustics, University of Oldenburg, Oldenburg, Germany

**Keywords:** timbre perception, music cognition, psychoacoustics, conceptual framework, definitions

## 1. Introduction

If there is one thing about timbre that researchers in psychoacoustics and music psychology agree on, it is the claim that it is a poorly understood auditory attribute. One facet of this commonplace conception is that it is not only the complexity of the subject matter that complicates research, but also that timbre is hard to define (cf., Krumhansl, 1989). Perhaps for lack of a better alternative, one can observe a curious habit in introductory sections of articles on timbre, namely to cite a definition from the American National Standards Institute (ANSI) and to elaborate on its shortcomings. For the sake of completeness (and tradition!) we recall:

“Timbre. That attribute of auditory sensation which enables a listener to judge that two nonidentical sounds, similarly presented and having the same loudness and pitch, are dissimilar [sic]. NOTE-Timbre depends primarily upon the frequency spectrum, although it also depends upon the sound pressure and the temporal characteristics of the sound.” (ANSI, 1994, p. 35)

One of the strongest criticisms of this conceptual framing was given by Bregman (1990), commenting,

“This is, of course, no definition at all. […] The problem with timbre is that it is the name for an ill-defined wastebasket category. […] I think the definition […] should be this: ‘We do not know how to define timbre, but it is not loudness and it is not pitch.’ […] What we need is a better vocabulary concerning timbre.” (pp. 92–93)

In an even more radical spirit, Martin (1999, p. 43) proposed, “[Timbre] is empty of scientific meaning, and should be expunged from the vocabulary of hearing science.” Almost 20 years later, although the notion is still part of the terminology, we are far from having reached a clearer taxonomy. One could even ask: Can something useful be done with the wastebasket in the end? In what follows, we propose four conceptual distinctions for timbre.

## 2. Timbre is a perceptual attribute

Already in the Nineteenth century, the title of Helmholtz's seminal treatise “On the sensations of tone as a physiological basis for the study of music” (von Helmholtz, 1885/1954) distinguishes an external physical sound event (the tone) from its internal perceptual representation (the sensation). The sensation comprises subjective auditory attributes such as pitch, loudness, and timbre, but the physical tone does not. Accordingly, the ANSI definition explicitly addresses sensory attributes. There are, unfortunately, many examples of a different type of usage, where timbre is primarily used to refer to features of physical sound events. These cannot only be found in adjacent academic disciplines such as music theory or music information retrieval, but even in music psychology, where the term is at times used as a shorthand for a sound event or complex tone, the relevant perceptual attribute of which is timbral in nature (e.g., “listeners were presented with three timbres”). This shorthand usage is tempting but harmful. It encourages the reader to equate the sound event and its timbre, which are in reality connected by a complex sequence of information-processing steps in the human auditory system. It becomes particularly problematic in conjunction with ecological views of perception, which often appear to circumvent the problem of information transformation by proclaiming a direct correspondence between perception and the world. As noted by Clarke (2005),

“The amplitude and frequency distribution of the sounds emitted when this piece of hollowed wood is struck are a direct consequence of the physical properties of the wood itself—are an ‘imprint’ of its physical structure—and an organism does not have to do complex processing to ‘decode’ the information within the source: it needs to have a perceptual system that will resonate to the information.” (p. 18)

A crux of the belief that the perceptual system is attuned to the “perceptual invariants” of the environment is, however, that “the detection of physical invariants, like image surfaces, is exactly and precisely an information-processing problem, in modern terminology” (Marr, 1982, p. 30). We need to study the ways in which auditory representations are robust to transformations of the acoustic signal given a specific context, in order to understand the correspondence of tone and sensation.

One can even observe more hazardous attempts to rephrase timbre as not primarily depending on perception. In a recent ANSI critique from a composer's viewpoint, Roads (2015) states,

“[The ANSI definition] describes timbre as a perceptual phenomenon, and not as an attribute of a physical sound. Despite this, everyone has an intuitive sense of timbre as an attribute of a sound like pitch or loudness (e.g., ‘the bassoon timbre’ […]). From a compositional point of view, we are interested in the physical nature of timbre […] in order to manipulate it for aesthetic purposes.” (p. xviii)

On the contrary, we insist that timbre is a *perceptual* attribute, as are pitch and loudness. Furthermore, there does not exist *the* bassoon timbre, but rather *a* bassoon timbre at a given pitch and dynamic, produced with a specific articulation and playing technique (see section 4). In order not to let the indispensable interdisciplinary discourse around timbre disintegrate into terminological incoherence, we should resist tempting shorthands right from the start and clearly separate physical sound events or tones and their morphologies (as well as their representations via musical scores, sampled time-pressure audio signals, spectrotemporal analyses, etc.) from the resulting auditory sensations. The three distinctions that follow consequently address timbre as a perceptual attribute.

## 3. Timbre is a quality and a contributor to source identity

There are two standard approaches in which timbre as a perceptual attribute is defined. Both approaches consider timbre as a bundle of auditory sensory features, to which, however, subtly different functions are ascribed. On the one hand, there is the (ANSI-like) definition by negation that encompasses all auditory attributes that allow listeners to perceive differences between sounds of equalized pitch, loudness, and say, spatial position. Here, the function of timbre attributes remains as vague as to allow listeners to engage in dissimilarity ratings and discrimination tasks. In this approach, timbre is referred to as quality: Two sounds can be declared qualitatively dissimilar without bearing semantic associations or without their source/cause mechanisms being identified. On the other hand, timbre is indeed defined via this latter role, namely as that collection of auditory sensory features that primarily contributes to the inference (or specification) of sound sources and events (although timbral differences do not always correspond to differences in sound sources, see below). Here the function ascribed to timbral attributes is tied to an identification task.

The difference between viewing timbre from the angles of qualitative comparison and source identification is not always clearly articulated. Dissimilarity studies that investigate timbre as qualia and work with acoustic stimuli may fail to account for the effects of source identification in dissimilarity ratings. In fact, the latent structure that underlies dissimilarity ratings is modeled by acoustic properties, implicitly assuming that dissimilarity ratings are solely based on the sensory representation of the sounds' acoustic features and not influenced by semantic categories elicited by the features of sound sources. It is questionable whether source identification can be neglected for acoustic stimuli, however, as one might argue that listeners “can't help” but integrate semantic information into dissimilarity ratings of Western orchestral instrument tones (Siedenburg et al., 2016b). In order not to conflate a study of sensory similarity with semantic factors, it is important to take into account the distinction between timbre as a quality and timbre as a contributor to source identity (also see Lemaitre et al., 2010).

## 4. Timbre functions on different scales of detail

When Helmholtz noted “By the quality of a tone [*Klangfarbe*] we mean that peculiarity which distinguishes the musical tone of a violin from that of a flute or that of a clarinet or that of the human voice, when all these instruments produce the same note at the same pitch” (von Helmholtz, 1885/1954, p. 10), he (perhaps unwittingly) provided the textbook definition of timbre for the next 150 years. This sentence operationalizes timbre via the perceptual differences based on the distinct acoustics of sound sources such as the flute and clarinet, and, like the ANSI definition, only compares timbre across tones with the same pitch, loudness, and duration.

Apart from the cul-de-sac in which this definition deprives any non-pitched sound of its timbre (Bregman, 1990, p. 92), the approach also neglects the fact that most pitched musical instruments can give rise to whole palettes of distinct timbral qualities which covary with pitch and loudness. Not only do different playing techniques and articulations affect physical and timbral properties of tones (e.g., Barthet et al., 2010), but a *fortissimo* comes with many pronounced partials (and a correspondingly bright timbre), whereas a *pianissimo* yields significantly attenuated amplitudes of higher order partials (Meyer, 1995). A tone's spectral content also covaries with fundamental frequency (*F*_0_) and playing effort. Low-pitched registers comprise many partial tones, higher tones do not. The acoustical covariance of *F*_0_ and spectrotemporal envelope shape appears to lead to small but systematic interactions between pitch and timbre (e.g., Marozeau and de Cheveigné, 2007), and these relations appear to be supported by perceptual learning (Sandell and Chronopoulos, 1997) and musical training (Steele and Williams, 2006). The corresponding pitch-timbre “covariance matrices” are likely to be used as a valuable perceptual cue for source identification (Handel and Erickson, 2004), although this research topic has been barely explored.

On an even more fine-grained scale, there can be differences between sounds from exemplars of the same type of sound-producing objects or algorithms (such as a Stradivarius violin and an inexpensive factory-made model). The ways in which this translates into audible timbral differences and how these relate to judged instrument quality (in the sense of good vs. bad) is yet another research topic (cf., Saitis et al., 2012).

In sum, it is misleading to suggest that one sound-producing object or instrument yields exactly one timbre. Contrary to parlance of “the bassoon timbre,” there is no single timbre that *fully* characterizes the bassoon. The timbre of a bassoon tone depends on pitch, playing effort, articulation, fingering, etc. In light of a biological analogy, a single type of sound-producing object or sound-synthesis algorithm may give rise to a timbral *genus* that can encompass various timbral *species*. These species may feature systematic variation along various parameters, such as playing technique, covariance with pitch and loudness, or expressive intent. Genera group into *families* (e.g., corresponding to the timbres from string vs. brass instruments) and at some point into *kingdoms* (timbres related to, say, acoustic vs. electronic means of sound production). Overall, this yields a “hierarchy of embedded distinctions” (Krumhansl, 1989, p. 45) that encompasses scales of different timbral detail to which the ANSI definition is agnostic and the textbook definition ignorant.

## 5. Timbre is a property of fused auditory events

Polyphonic music is the unequivocal target territory for timbre research. Consequently, studies are beginning to explore the acoustic correlates of what has been called “polyphonic timbre” (Alluri and Toiviainen, 2010), “capturing the overall emerging timbre of a jazz ensemble, a rock concert, or a symphony,” and thus the “global sound” of a piece of music.

Analogous to pitch and loudness, however, we view timbre as a perceptual property of perceptually fused auditory events. If two or more auditory events do not fuse, they do not contribute to the same timbre. Sounds from a bass-drum, a handclap, and a synth pad usually do not fuse into a single auditory image, such that each of these sounds will possess an individual timbre in the mind of a listener. It is the emergent property of the combination of the individual timbres that evokes hip-hop, but there is no a unitary “hip-hop timbre.”

In fact, auditory scene analysis (ASA) principles do not provide a definitive borderline of where segregation ends, because stream formation depends on the listener's focus in the ASA hierarchy. Not entirely fused (heterogeneous) musical lines can be heard as one stream or many, depending on auditory focus and musical context. On the other hand, completely disregarding ASA processes by extracting features from the audio mixture may contribute to the reported limitations in using music information retrieval algorithms as perceptual models (cf., Siedenburg et al., 2016a). As perhaps best summarized by Aucouturier and Pachet (2007, p. 659),

“Overall, this suggests that the horizontal coding of frames of data, without any account of source separation and selective attention, is a very inefficient representation of polyphonic musical data, and not cognitively plausible.”

A metaphor might drawn from the relation between pitch and harmony perception, where one can still hear individual pitches (timbres), but there is another quality that emerges from the relations among the pitches (timbres). Hence, rather than presupposing that polyphonic music gives rise to unitary auditory images (which the notion of “polyphonic timbre” suggests), we believe that it is the combinatorial interplay of timbres that is at the heart of the perception of polyphonic music.

## 6. Conclusion

By proposing four basic distinctions for the notion of timbre we hope to clear up some confusion around what has been claimed to be the terminological wastebasket of music psychology and psychoacoustics—musical timbre. In direct opposition to physical realists such as Isaac (2017), we propose to locate timbre on the perceptual side of the “psychophysical divide,” i.e., in the mind of the listener instead of in physical properties. We further argue that the notion is commonly viewed from different angles: as qualia and as a contributor to source identity, but the language around this distinction needs to be clarified to avoid confusion between them. We have illustrated that there may be large- or small-scale timbral differences (e.g., arising from timbral families vs. species), and that timbre is a property of fused auditory events instead of multi-stream auditory mixtures. We do not claim that this is an exhaustive categorization—more fine-grained taxonomies must be developed in order to account for timbre's perceptual richness. Nonetheless, the four proposed distinctions may serve as a basic taxonomy to clarify discourse in future inquiries into timbre. Furthermore, each distinction encompasses its own host of research questions that subsequent empirical work may address. In any case, once a few layers of dust are removed, what we had thought of as a wastebasket turns out to be a colorful umbrella(-term) upside down.

The composer Manoury (1991) observed that “One of the most striking paradoxes concerning timbre is that when we knew less about it, it didn't pose much of a problem” (p. 293). This can also be put in more optimistic terms: We already know much about timbre. We understand its plentiful, distinct colors are real, and they won't go away. It is time to let inadequate standards rest and start to focus on the specifics.

## Author contributions

KS and SM discussed ideas relevant to the topic. KS devised the first draft of the manuscript, which subsequently underwent several substantial revisions based on joint discussion among the co-authors.

### Conflict of interest statement

The authors declare that the research was conducted in the absence of any commercial or financial relationships that could be construed as a potential conflict of interest.
